# Concomitant Swine Influenza A Virus Infection Alters PRRSV1 MLV Viremia in Piglets but Does Not Interfere with Vaccine Protection in Experimental Conditions

**DOI:** 10.3390/vaccines9040356

**Published:** 2021-04-07

**Authors:** Patricia Renson, Céline Deblanc, Juliette Bougon, Mireille Le Dimna, Stéphane Gorin, Sophie Mahé, Nicolas Barbier, Frédéric Paboeuf, Gaëlle Simon, Olivier Bourry

**Affiliations:** 1 Agence Nationale de Sécurité Sanitaire de l’Alimentation, de l’Environnement et du Travail (Anses), Unité Virologie Immunologie Porcines, BP 53, 22440 Ploufragan, France; celine.deblanc@anses.fr (C.D.); juliette.bougon@anses.fr (J.B.); mireille.ledimna@anses.fr (M.L.D.); stephane.gorin@anses.fr (S.G.); sophie.mahe@anses.fr (S.M.); nicolas.barbier@anses.fr (N.B.); gaelle.simon@anses.fr (G.S.); olivier.bourry@anses.fr (O.B.); 2 Université de Rennes (UNIR), Cité Internationale, 1 Place Paul Ricoeur, CS 54417, 35044 Rennes, France; 3 Anses, Service de Production de Porcs Assainis et d’Expérimentation, BP 53, 22440 Ploufragan, France; frederic.paboeuf@anses.fr

**Keywords:** porcine reproductive and respiratory syndrome, swine influenza A virus, modified-live vaccine, interference, interferon

## Abstract

Modified-live vaccines (MLVs) against porcine reproductive and respiratory syndrome viruses (PRRSVs) are usually administrated to piglets at weaning when swine influenza A virus (swIAV) infections frequently occur. SwIAV infection induces a strong interferon alpha (IFNa) response and IFNa was shown to abrogate PRRSV2 MLV replication and an inherent immune response. In this study, we evaluated the impacts of swIAV infection on the replication of a PRRSV1 MLV (MLV1), post-vaccine immune responses and post-challenge vaccine efficacy at both the systemic and pulmonary levels. Piglets were either swIAV inoculated and MLV1 vaccinated 6 h apart or singly vaccinated or mock inoculated and mock vaccinated. Four weeks after vaccination, the piglets were challenged with a PRRSV1 field strain. The results showed that swIAV infection delayed MLV1 viremia by six days and post-vaccine seroconversion by four days. After the PRRSV1 challenge, the swIAV enhanced the PRRSV1-specific cell-mediated immunity (CMI) but the PRRSV1 field strain viremia was not better controlled. High IFNa levels that were detected early after swIAV infection could have been responsible for both the inhibition of MLV1 replication and CMI enhancement. Thus, whereas swIAV infection had a negative impact on humoral responses post-vaccination, it did not interfere with the protective effectiveness of the PRRSV MLV1 in our experimental conditions.

## 1. Introduction

Porcine reproductive and respiratory syndrome (PRRS) is a viral disease of swine with huge economic impact worldwide, characterized by reproductive failures in pregnant sows and respiratory disorders and growth retardation in piglets [1,2]. The disease is caused by PRRS viruses (PRRSVs), which are single-strand positive sense-RNA viruses that belong to the *Arteriviridae* family and exist as two different species originating from Europe for the PRRSV1 or from North America for the PRRSV2 (recently re-named as *Betaarterivirus suid 1* and *2*, respectively) [3,4]. Nowadays, both species are distributed worldwide but only PRRSV1 strains are circulating in France.

To control PRRS, modified-live vaccines (MLVs) are frequently used in sows or young piglets but only partial protection is achieved in the field, mainly reducing clinical signs by 70% in piglets [5]. In experimental conditions, the efficacy of PRRSV MLVs was demonstrated in specific pathogen free (SPF) piglets, reducing the viremia of the challenge strain in inoculated animals and almost completely preventing its transmission to sentinel piglets [6]. The low efficacy of PRRSV MLVs shown under field conditions may be related to different factors. Thus, we previously demonstrated that maternally-derived neutralizing antibodies can decrease both the post-vaccination immune response and the PRRSV MLV1 efficacy [7,8]. Moreover, we suggested that viral infections, through interferon alpha (IFNa) secretion, could also affect the efficacy of the PRRSV MLV1 in the field [8].

IFNa is a cytokine of the innate immune response mainly induced after viral infections. In piglets, high levels of IFNa were detected during infections by coronaviruses such as porcine respiratory coronavirus (PRCoV) and transmissible gastroenteritis coronavirus (TGEV) and by rotavirus or by influenza A viruses (swIAVs) [9,10,11]. IFNa has an anti-viral activity against a broad spectrum of viruses and the PRRSV is particularly sensitive to the IFNa effect [12,13]. In response, the PRRSV has developed mechanisms to inhibit IFNa induction so that the PRRSV infection itself is generally characterized by low levels of IFNa [14,15].

The effect of IFNa on the PRRSV MLV or field strains has been studied to some extent. In 2017, Brockmeier et al. showed that delivering IFNa through an adenoviral vector (Ad5-pIFNa) completely inhibited the replication of a PRRSV2 MLV (MLV2) and abrogated the induction of the post-vaccine immune response [16]. Focusing on viruses with respiratory tropism as a PRRSV, Buddaert et al. showed decreased PRRSV1 titers in the lungs of pigs that were previously infected by PRCoV two days earlier compared with singly PRRSV1-infected pigs [12].

As major ethological agents of the porcine respiratory disease complex (PRDC), swIAVs and PRRSVs frequently co-circulate in pig herds and potential interactions between both viruses are very likely [17]. In French pig herds, an infection with the H1N2 but not the H1N1 swIAV was found to be associated with a PRRSV infection [18]. In France, the swIAV serological prevalence at the herd level was evaluated at 48.8% [19] and a high frequency of swIAV-positive herds was reported by the RESAVIP surveillance network among herds with respiratory disorders, with relatively stable levels from 2012 to 2018 ranging from 41.8% to 53.6% [20]. SwIAVs can circulate in piglets at weaning [21,22] even in the presence of maternally-derived antibodies (MDA) [23,24]. Furthermore, experimental infections showed that high IFNa levels were early detected in the serum and bronchoalveolar lavage fluid of swIAV-infected piglets both for H1N1 [11] and H1N2 virus subtypes [25] with a peak at 24 h post-inoculation.

Considering that the PRRSV MLV vaccination of piglets mainly takes place at weaning and that this period is also favorable to swIAV infections, a viral infection associated with high IFNa production, it appears interesting to investigate the possible interaction between swIAV infection and the PRRSV MLV vaccination. The objectives of the present study were to evaluate in SPF piglets the impact of an H1N2 swIAV infection on (i) the replication of a PRRSV1 MLV (MLV1) and (ii) the induction of the post-vaccination immune response, investigating the potential role of IFNa in these interactions. Finally, (iii) the effect of the swIAV infection on the vaccine efficacy was assessed towards a PRRSV1 challenge.

## 2. Materials and Methods

### 2.1. Viruses and Vaccines

The H1N2 swIAV A/Sw/France/Ille et Vilaine/0415/2011 strain is a human-like reassortant swine (GenBank accession No. KR699787-94) isolated from nasal swabs taken from pigs with acute respiratory disease and propagated and titrated in Madin–Darby canine kidney (MDCK) cells for five passages.

The Porcilis PRRS vaccine (MSD, Beaucouzé, France; batch No. A213BB01) was used in the in vivo experiment as the MLV1 vaccine. For in vitro analyses, the MLV1 vaccine strain was obtained by suspending the lyophilized vaccine in Eagle’s minimal essential medium (EMEM), propagating and titrating it on MARC-145 cells for two and three passages for virus neutralization tests and for ELISPOT analyses, respectively.

The PRRSV1 “Finistere” strain, referenced as PRRS-FR-2005-29-24-1 (GenBank accession No. KY366411), was isolated in France in 2005 from a herd with reproductive failures in sows and propagated and titrated in porcine primary alveolar macrophages for two passages for animal inoculations and for six passages for ELISPOT analyses.

The H1N2 swIAV and PRRSV1 Finistere inocula were prepared by diluting each strain in EMEM.

### 2.2. Animal Experiment

**Figure 1:**
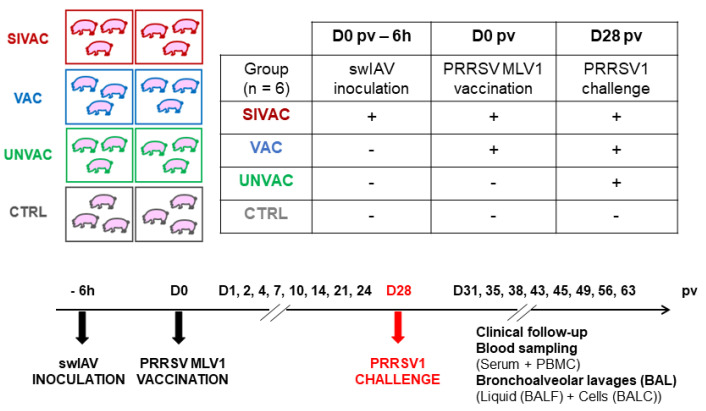
Description of the experiment design.

Twenty-four 5-week-old SPF large white piglets from Anses-Ploufragan protected animal facilities were randomly distributed to four groups according to parental origin, weight and gender (Figure 1). At day 0 post-vaccination (D0 pv), piglets were either inoculated with 10^6^ TCID_50_ per pig (in 5 mL) of swIAV by an endotracheal route using a nasogastric probe of 4.5 mm of diameter and 50 cm long (SIVAC group) or mock inoculated with an EMEM cell culture medium (VAC, UNVAC and CTRL groups). Six hours later, piglets were either vaccinated using one dose of the MLV1 (2 mL) by intramuscular injection in the neck (SIVAC and VAC groups) or mock vaccinated with vaccine diluent (UNVAC and CTRL groups).

At D28 pv, piglets were either challenged with 5 × 10^5^ TCID_50_/pig (in 5 mL) of the PRRSV1 Finistere strain by intranasal inoculation (2.5 mL per nostril) (SIVAC, VAC and UNVAC groups) or mock inoculated with an EMEM cell culture medium (CTRL group).

The rectal temperature, daily weight and clinical score were monitored daily using a template adapted from Deblanc et al. [25]. A temperature higher than 40 °C was reported as hyperthermia. Serum samples were collected at D0, D1, D2, D4, D7, D10, D14, D21, D24, D31, D35, D38, D43, D45, D49, D56 and D63 pv. Peripheral blood mononuclear cells (PBMC) were purified from heparinized blood at D0, D14, D24, D43 and D56 pv by Ficoll-paque gradient density separation using LeucoSep tubes (Greiner Bio-One, Kremsmünster, Austria).

Bronchoalverolar lavages (BALs) were performed under general anesthesia using an intramuscular injection of 10 mg/kg de Zoletil^®^ (Virbac, Carros, France) at D0, D1, D4, D7, D10, D14, D21, D24, D43 and D56 pv by infusing 2 × 20 mL of sterile PBS instilled in the lungs through a nasogastric probe of 4.5 mm of diameter and 50 or 57 cm long depending on the age of the animals (Laboratoires Euromedis, Neuilly-sous-Clermont, France). The BAL cells (BALCs) were separated from the BAL fluid (BALF) by centrifugation.

In addition, nasal swabs were sampled at D0, D1, D2, D4, D7 and D10 pv on piglets from the SIVAC group in order to identify potential lesions (especially in the lungs).

From D70 to D73 pv (D42 to D45 post-challenge (pc)), the animals were euthanized and a necropsy was conducted on each pig.

All experiments were authorized by the French Ministry for Research (project No. APAFIS#14266-2018032615007483v3) and approved by the national ethics committee number 16.

### 2.3. Viral Quantifications

The PRRSV1 genome from the serum and the BALF as well as the swIAV genome from the BALF were purified using the NucleoSpin 8 virus kit according to the manufacturer’s instructions (Macherey-Nagel, Düren, Germany). The SwIAV genome was isolated from nasal swab supernatants using the NucleoSpin 8 RNA kit (Macherey-Nagel, Düren, Germany).

Before the challenge, the MLV1 strain genome was detected using the in-house pan-PRRSV qRT-PCR described by Charpin et al. [26]. After the challenge, the quantification of the Finistere strain genome was assessed by a specific qRT-PCR as previously described [27]. Briefly, PRRSV genomic RNA was further amplified using the SuperScript III platinum one-step qRT-PCR kit (Life Technologie, Carlsbad, CA, USA) with probes and primers, targeting either the ORF7 of the PRRSV1 (forward: 5′-AACGYTCCCTCTGCTTGC-3′, reverse: 5′-CTCAACCTGAAAACTGACCTTCC-3′, probe: 6FAM-CGATCCAGACGGCTTTYAATCAAGGCG-TAM) or the ORF5 of the Finistere strain (forward: 5′-TATGCGAGCTGAATGGGACC-3′, reverse: 5′-AGGATATGAGTGGCAACCGG-3′, probe: 6FAM-TGGGCAGTTGAGACTTTCGTGCT-TAM). The PRRSV RT-PCR was conducted in duplex with the amplification of the porcine beta-actin gene as the internal control (forward: 5′-CTCGATCATGAAGTGCGACGT-3′, reverse: 5′-GTGATCTCCTTCTGCATCCTGTC-3′, probe: TET-ATCAGGAAGGACCTCTACGCCAACACGG-BHQ1). The PRRSV genomic loads in sera and BALF samples were quantified using a standard viral range of either the vaccine strain or the Finistere strain (with known infectious titers) diluted in a corresponding biological matrix collected from SPF pigs. The results were expressed as equivalent (eq) TCID_50_/mL of the type of sample used.

SwIAV viral loads were quantified by RT-qPCR targeting the M gene as previously described [24]. Briefly, swIAV genomic RNA was further amplified using the GoTaq^®^ Probe 1-Step RT-qPCR System (Promega, Madison, WI, USA) with probes and primers targeting the M gene (forward: 5′-AGATGAGTCYTCTAACCGAGGTCG-3′, reverse: 5′-TGCAAARACAYYTTCMAGTCTCTG-3, probe: 6FAM-TCAGGCCCCCTCAAAGCCGA-BHQ1). As for the PRRSV amplifications, the porcine beta-actin amplification was performed in duplex. The results were expressed as the M gene copy number per 10^6^ copies of the beta-actin gene for nasal swab supernatants or per ml of BALF.

For all viral genomic quantifications, samples with Ct values under the last dilution of the viral range were assigned to an extrapolated viral load and were included in the mean calculation.

### 2.4. Antibody Measurements

Antibodies against the N protein of the PRRSV were detected in serum using BioLisa PRRSV Ab ELISA (BioSellal, Dardilly, France) according to manufacturer’s instructions. For detection in BALF, the ELISA’s protocol was adapted using a sample starting dilution of 1:2. Sample-to-positive (S/P) ratios with values equal to or higher than 0.4 were considered positive.

Neutralizing antibodies against the PRRSV were titrated by a virus neutralization test on MARC-145 cells using the MLV1 strain as previously described [7].

Antibodies against the nucleoprotein of the swIAV were detected in serum using ID Screen Influenza A Ab competition multi-species ELISA (ID.Vet, Grabels, France) as described in the manufacturer’s instructions. In BALF, the protocol was modified starting from undiluted samples. Inhibition percentages with values equal to or lower than 45% were considered positive.

### 2.5. Cell-Mediated Immune Response (CMI) Assessment

PRRSV-specific IFNg-secreting cells (IFNg-SCs) were quantified in triplicate by ELISPOT in blood or in BAL as described by Fablet et al. [7] after 16 h stimulation of 4 × 10^5^ PBMC or 5 × 10^5^ BALC with either the MLV1 strain or the Finistere strain (obtained as described above) at a multiplicity of infection of 0.2. In addition, each sample was stimulated in triplicate with culture media or 10 μg/mL of PHA (Eurobio, Les Ulis, France) as controls. The stimulation of PBMC or BALCs was performed on MultiScreen-IP 0.45 µm nitrocellulose plates (Millipore, Burlington, MA, USA), previously coated overnight at 4 °C with 500 ng/well of an anti-pig IFNg monoclonal antibody (mAb) (clone P2G10, BD Biosciences, San Jose, CA, USA). After cell stimulation, the secreted IFNg was visualized by incubating plates for 2 h at room temperature (RT) with 25 ng/well of a biotinylated anti-pig IFNg mAb (clone P2C11, BD Biosciences, San Jose, CA, USA) followed by incubation for 1 h at RT in streptavidin alkaline phosphatase (1:1000 dilution, Caltag Medsystems, Buckingham, UK) then for 20 min at RT in alkaline phosphatase substrate kit reagents (Bio-Rad, Hercules, CA, USA). The number of spots per well was counted using an ImmunoSpot S5 UV Analyzer (CTL, Shaker Heights, OH, USA). The results were reported as numbers of IFNg-SCs per million of PBMC or BALC.

### 2.6. Cytokine Measurements

Porcine IFNa was detected in serum or BALF using an in-house sandwich ELISA as described by Jamin et al. [28]. Briefly, Nunc-Immuno MaxiSorp surface plates (Nunc, Roskilde, Denmark) were coated overnight at RT with an anti-pig IFNa mAb (clone K9, PBL Assay Science, Piscataway, NJ, USA) at 7.5 μg/mL in PBS then blocked for 1 h at 37 °C in PBS containing 5% BSA and 0.05% Tween 20. Serum samples and a standard range of porcine recombinant IFNa (PBL Assay Science, Piscataway, NJ, USA) were incubated for 2 h at RT. Biotinylated-IFNa mAb (1:200 dilution, clone F17, PBL Assay Science, Piscataway, NJ, USA; Lynx rapid biotin type1 antibody conjugation kit, Bio-Rad, Hercules, CA, USA) was then added for 2 h at RT and peroxidase-conjugated ExtrAvidin (1:1000 dilution, Sigma–Aldrich, Saint-Louis, MO, USA) for 1 h at 37 °C. The peroxidase activity was measured using TMB substrate (Sigma–Aldrich, Saint-Louis, MO, USA) at 450 nm.

Porcine interleukine 10 (IL10) and interferon gamma (IFNg) gene expressions were measured in BALCs using a TaqMan-based triplex real-time RT-PCR described by Petrov et al. that used both b-actin (forward: 5′-AGCGCAAGTACTCCGTGTG-3′, reverse: 5′-CGGACTCATCGTACTCCTGCTT-3′, probe: HEX-TCGCTGTCCACCTTCCAGCAGATGT-BHQ1) and GAPDH (forward: 5′ACATGGCCTCCAAGGAGTAAGA-3′, reverse: 5′-GATCGAGTTGGGGCTGTGACT-3′, probe: TXR-CCACCAACCCCAGCAAGAGCACGC-BHQ1) as endogenous controls to normalize the cytokine gene expression [29]. Petrov’s RT-PCR was performed with a modified protocol using the SuperScript III Platinum One-Step Quantitative RT-PCR System (Life Technologies, Carlsbad, CA, USA) and previously published primers and probes by Royaee et al. for IL10 (forward: TGAGAACAGCTGCATCCACTTC, reverse: TCTGGTCCTTCGTTTGAAAGAAA, probe: 6FAM-CAACCAGCCTGCCCCACATGC-BHQ1) and IFNg (forward: TGGTAGCTCTGGGAAACTGAATG, reverse: GGCTTTGCGCTGGATCTG, probe: 6FAM-CTTCGAAAAGCTGATTAAAATTCCGGTAGATAATCTGC-BHQ1) [30]. The results were expressed as relative expressions of target genes, calculated using the R = 2^−ΔΔCt^ equation with regard to the mean expression level in the BALCs of UNVAC pigs collected at the respective time point.

### 2.7. Statistical Analyses

All data and calculated areas under the curve (AUC) were compared between groups applying the Kruskal–Wallis test with Holm’s post-hoc pairwise comparisons using R software 3.4.4 with the limit of the significance of the *p*-value < 0.05. On figures, different letters indicated the significant differences obtained by comparing groups either with the CTRL group (a) or with the UNVAC group (b) or between the VAC and SIVAC groups (c).

## 3. Results

### 3.1. Clinical Follow-Up during the Post-Vaccination Phase

**Figure 2:**
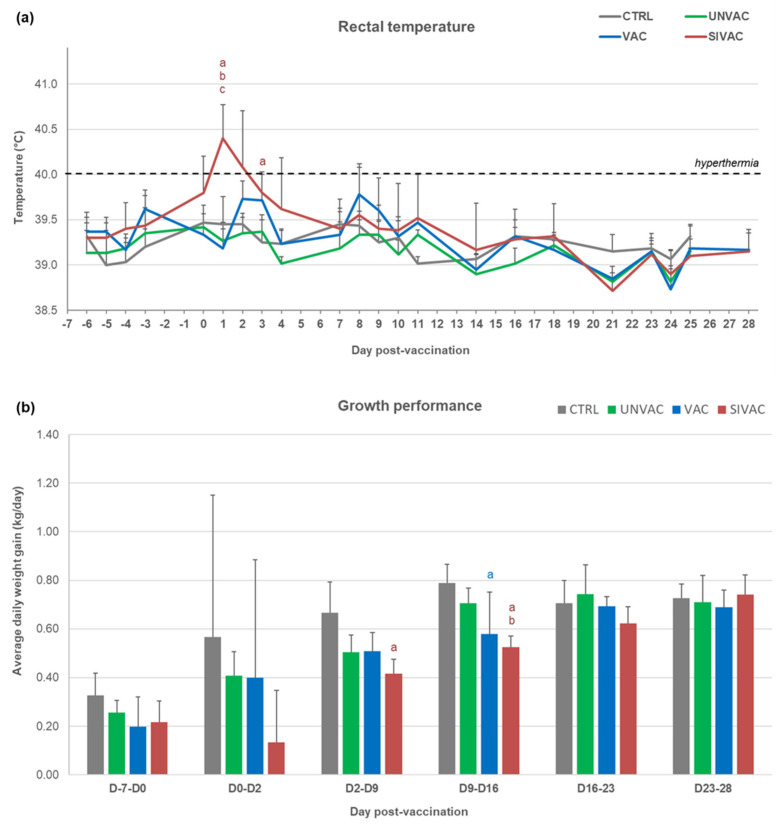
Clinical data recorded during the post-vaccination period (D0 to D28 pv). (**a**) Rectal temperature; (**b**) Average daily weight gain of piglets. All data are reported as the mean (±SD) of the results obtained from piglets in each group. Different letters (a–c) indicate that the considered group (specified by its color) was significantly different from the CTRL group (a), from the UNVAC group (b) or from the VAC group (c) with *p* < 0.05.

After MLV1 vaccination, a peak of hyperthermia (40.4 ± 0.4 °C) was recorded only in the SIVAC group at D1 pv (Figure 2a). Compared with CTRL piglets, the average daily weight gains (ADWGs) were significantly reduced between D2 and D16 pv and between D9 and D16 pv for SIVAC and VAC piglets respectively (Figure 2b).

### 3.2. SwIAV Infection Assessment in the Post-Vaccination Period

**Figure 3:**
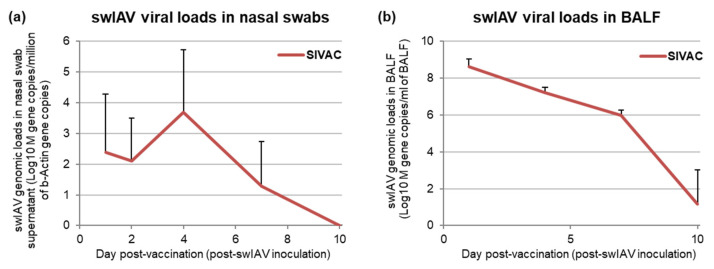
Swine influenza A virus (SwIAV) genomic loads quantified by M gene RT-qPCR (**a**) in nasal swab supernatants or (**b**) in bronchoalverolar lavage fluid (BALF) collected from piglets in the SIVAC group. Data are reported as the mean (±SD) of the group.

The swIAV genome was simultaneously detected as early as D1 pv (D1 post-swIAV inoculation) in nasal swab supernatants and BALF and until D7 and D10 pv in nasal swab supernatants and BALF, respectively (Figure 3). In BALF, viral loads were still high at D7 pv (5.97 ± 0.3 log10 M gene copies/mL of BALF). Antibodies against swIAV were detected simultaneously in serum and BALF from D7 pv, confirming all SIVAC animals had been swIAV-infected.

**Figure 4:**
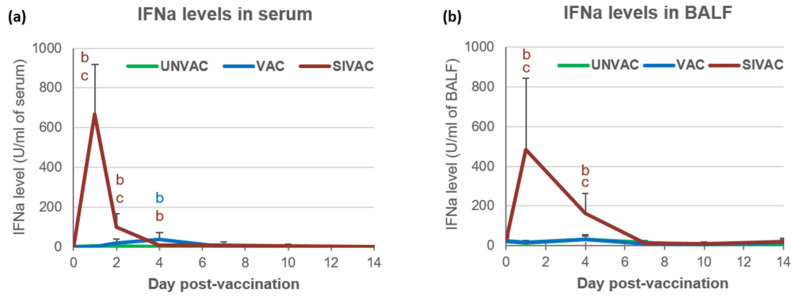
Interferon alpha (IFNa) levels quantified in (**a**) serum or (**b**) BALF. Data are reported as the mean (±SD) of the results obtained from all piglets in each group. Different letters (b,c) indicate that the considered group (specified by its color) was significantly different from the UNVAC group (b) or from the VAC group (c) with *p* < 0.05.

Quantification of IFNa in SIVAC piglets showed high levels in serum and BALF at D1 pv (D1 post-swIAV inoculation) with 668 ± 251 U/mL and 484 ± 360 U/mL, respectively (Figure 4). IFNa concentrations then decreased fast until being undetectable at D7 pv. In the VAC group, IFNa was only detected at D4 pv in serum at a very low level (36 ± 37 U/mL), which resulted in significant differences between the SIVAC and VAC groups at D1 and D2 pv in serum and at D1 and D4 pv in BALF.

### 3.3. IFNg and IL10 Gene Expression in BALCs in the Post-Vaccination Period

**Figure 5:**
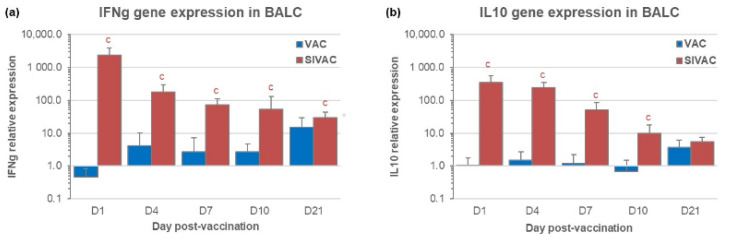
Relative gene expression assessed by RT-PCR in BALCs from the SIVAC and VAC groups for (**a**) IFNg or (**b**) IL10 using expression results obtained in the UNVAC group as the reference. Data are reported as the mean (±SD) of the results obtained from all piglets in each group. Letter (c) indicates that the SIVAC group was significantly different from the VAC group with *p* < 0.05.

In the post-vaccination period (before the PRRSV1 challenge), the gene expressions of IFNg and IL10 were quantified by RT-PCR in the BALCs of the SIVAC and VAC groups (Figure 5). The results showed an upregulation of both cytokines in the SIVAC group from D1 to D21 pv compared with the expression recorded in the UNVAC group at the same dates. The highest increased expressions were observed at D1 pv in the VAC group compared with the basal expression of the UNVAC piglets. Both cytokine gene expressions were not or only slightly upregulated before D21 pv, displaying a significant difference between the SIVAC and VAC groups from D1 to D10 pv for the expression of the IFNg gene as well as the IL10 gene (on average 10 to 100-fold higher for the SIVAC group). At D21 pv, the difference between the SIVAC and VAC groups was still significant for the IFNg expression only (*p* = 0.045).

### 3.4. MLV1 Viremia and the PRRSV Immune Response Assessment during the Post-Vaccination Period

**Figure 6:**
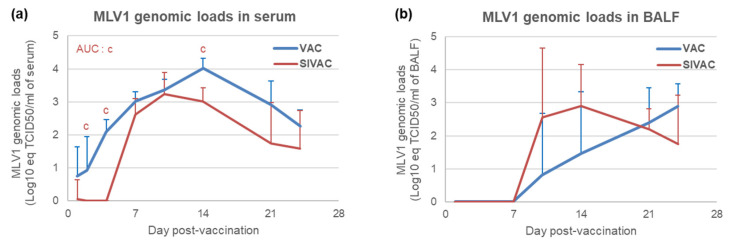
Porcine reproductive and respiratory syndrome virus (PRRSV) genomic loads in (**a**) serum or in (**b**) BALF. All data are reported as the mean (±SD) of the results obtained from all piglets in each group. Letter (c) indicates that the SIVAC group was significantly different from the VAC group with *p* < 0.05. AUC: area under the curve.

In the VAC group, the MLV1 genome was detected from D1 pv in all piglets. In the SIVAC group, it was detected at very low levels in the sera of two out of six piglets at D1 pv (under the limit of quantification) and remained undetectable in all animals until D7 pv when the viremia was first quantified (6/6 piglets) (Figure 6a). The viremia peak was reached at D14 pv for the VAC group (4.01 ± 0.31 log10 eq TCID_50_/mL of serum) and at D10 pv for the SIVAC pigs (3.02 ± 0.40 log_10_ eq TCID_50_/mL of serum). When comparing the two vaccinated groups over the post-vaccination period, the MLV1 viremia was significantly lower in the SIVAC group (*p* = 0.011 for comparisons of AUCs). In BALF, the vaccine genomic loads were quantified from D10 pv in two out of six VAC piglets and in four out of six SIVAC piglets, with no significant difference between the groups over the post-vaccination period (*p* = 0.112 for comparisons of AUCs) (Figure 6b).

**Figure 7:**
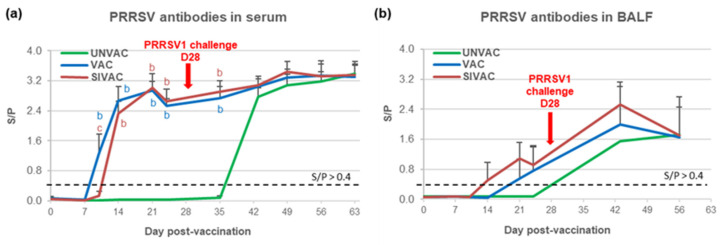
Anti-PRRSV antibody response assessed by ELISA in (**a**) serum or in (**b**) BALF. Data are reported as the mean (±SD) of results the obtained from all piglets in each group. Different letters (b,c) indicate that the considered group (specified by its color) was significantly different from the UNVAC group (b) or from the VAC group (c) with *p* < 0.05.

Serum antibodies directed against the PRRSV appeared at D14 pv for SIVAC animals and at D10 pv for VAC piglets (Figure 7a) with a significantly lower ELISA S/*p*-value for the SIVAC group at D10 pv. In BALF, the PRRSV antibodies were detected from D14 pv in three out of six SIVAC pigs and from D21 pv in three out of six VAC pigs without a significant difference between either group (Figure 7b).

**Figure 8:**
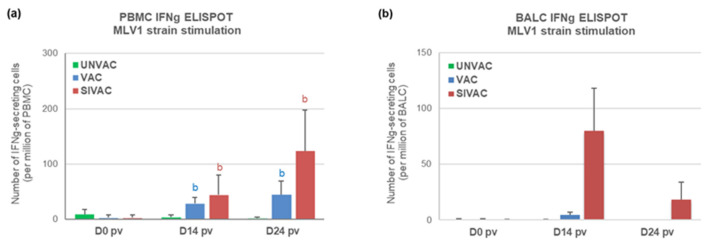
Anti-PRRSV cell-mediated immune response to the vaccine strain assessed by IFNg ELISPOT in (**a**) PBMC or in (**b**) BALCs. Data are reported as the mean (±SD) of the results obtained from all piglets in each group. Different letters (b,c) indicate that the considered group (specified by its color) was significantly different from the UNVAC group (b) or from the VAC group (c) with *p* < 0.05. There is no significance c found in these graphs.

In blood, cellular immunity specific to the MLV1 strain (assessed by IFNg ELISPOT), was observed from D14 pv in both the SIVAC and VAC groups (Figure 8a). At D24 pv, the number of IFNg-SCs tended to be higher for SIVAC piglets (124 ± 74 and 44 ± 25 IFNg-SCs per million of PBMC for the SIVAC and VAC groups, respectively; *p* = 0.093). Similarly, in lungs, IFNg-SCs were only detected in BALCs from the SIVAC group (3/6 piglets at D14 pv and 1/6 at D24 pv) without a significant statistical difference between the SIVAC and VAC groups (Figure 8b).

### 3.5. Evaluation of MLV1 Vaccine Efficacy after the PRRSV1 Challenge

**Figure 9:**
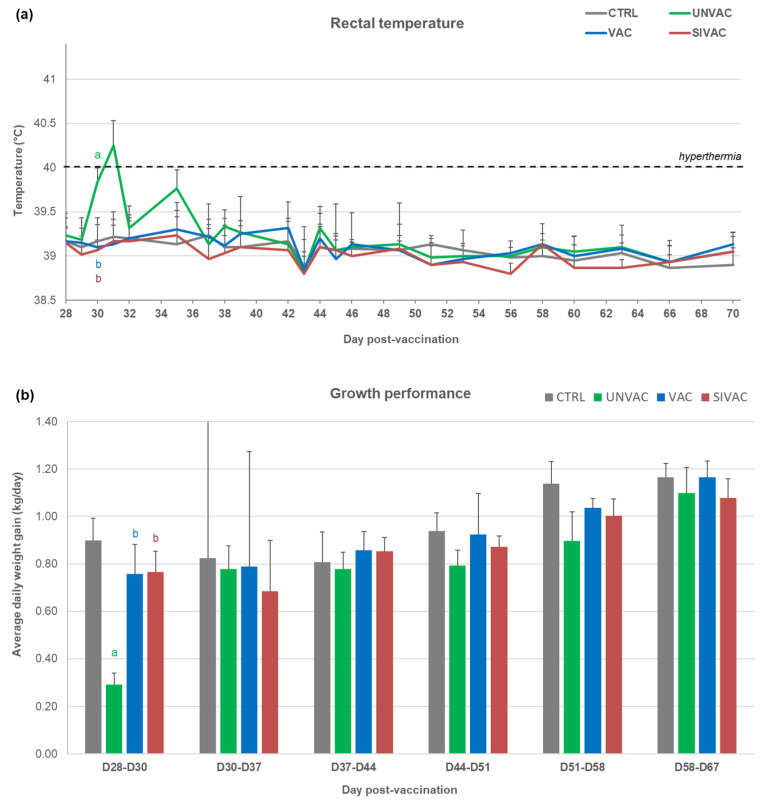
Clinical data recorded during the post-challenge period (D28 to D70 pv). (**a**) Rectal temperature; (**b**) Average daily weight gain of piglets. All data are reported as the mean (±SD) of the results obtained from piglets in each group. Different letters (a–c) indicate that the considered group (specified by its color) was significantly different from the CTRL group (a), from the UNVAC group (b) or from the VAC group (c) with *p* < 0.05. There is no significance c found in these graphs.

After the PRRSV1 challenge, hyperthermia was detected only in piglets from the UNVAC group at D30 (D2 pc) and D35 pv (D5 pc) with a significant increase of mean rectal temperature in this group at D30 pv compared with the other groups (Figure 9a). Regarding growth performances, the ADWG was decreased in the UNVAC group between D28 and during the post-challenge period; no respiratory clinical signs were noticed and no pulmonary lesions were observed at necropsies performed from D70 to D73 pv, corresponding with the D70–73 post-swIAV or the D42–45 post-PRRSV1 challenge.

**Figure 10:**
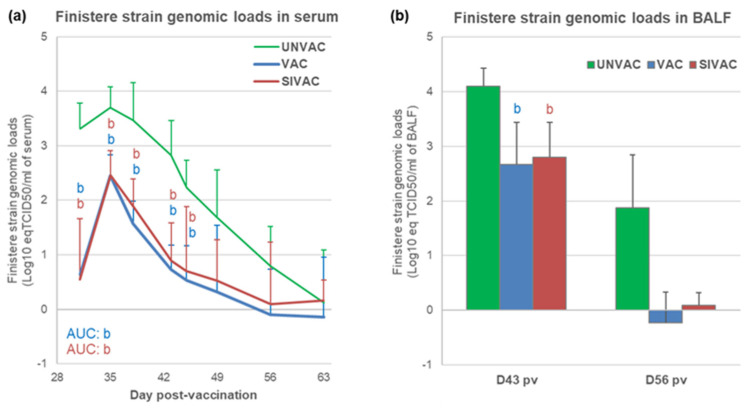
PRRSV1 Finistere strain genomic loads in (**a**) serum or in (**b**) BALF. Data are reported as the mean (±SD) of the results obtained from all piglets in each group. Different letters (b,c) indicate that the considered group (specified by its color) was significantly different from the UNVAC group (b) or from the VAC group (c) with *p* < 0.05. AUC: area under the curve. There is no significance c found in these graphs.

After the PRRSV1 challenge (at D28 pv), the quantification of the Finistere strain genomic load was assessed by a specific qRT-PCR. Compared with the UNVAC group, both the SIVAC and VAC groups displayed a significant reduction in challenge strain viremia over the entire post-challenge period (*p* ≤ 0.001 for both comparisons of AUCs) with lower mean viremia peaks at D35 pv (D7 pc) (2.46 ± 0.45, 2.45 ± 0.38 and 3.70 ± 0.39 log10 eq TCID_50_/mL for SIVAC, VAC and UNVAC groups, respectively) (Figure 10a). Finistere strain genomic loads in sera from SIVAC pigs were never different from those from VAC pigs.

Similar results were obtained in BALF (Figure 10b). Viral genomic loads were significantly reduced at D43 pv (D15 pc) for both the SIVAC and VAC groups compared with the UNVAC group (*p* = 0.006 and *p* = 0.008 for SIVAC and VAC, respectively).

**Figure 11:**
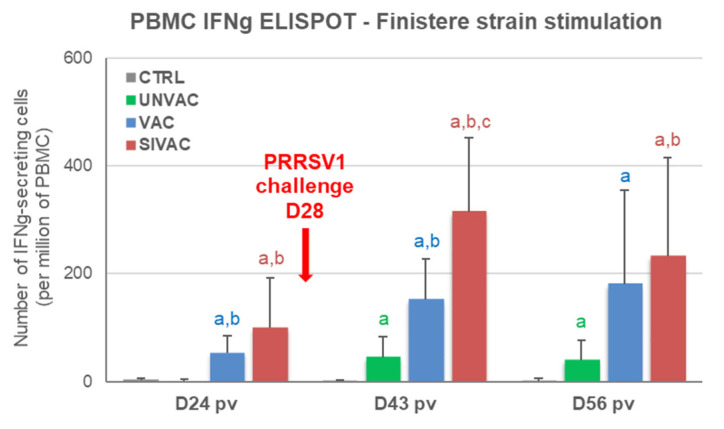
Anti-PRRSV cell-mediated response to the Finistere strain assessed by IFNg ELISPOT in peripheral blood mononuclear cells (PBMC). Data are reported as the mean (±SD) of the results obtained from all piglets in each group. Different letters (a–c) indicate that the considered group (specified by its color) was significantly different from the CTRL group (a), from the UNVAC group (b) or from the VAC group (c) with *p* < 0.05.

Regarding the immune response induced after the PRRSV1 challenge, antibodies were detected from D43 pv (D15 post-challenge) in sera and BALF from UNVAC pigs at levels equivalent to those measured for the SIVAC and VAC groups (Figure 7). These results were confirmed by titrating the PRRSV neutralizing antibodies in sera. Concerning the cell-mediated immunity specific to the Finistere strain, significantly higher levels of IFNg-SCs were detected in the blood of both the VAC and SIVAC groups compared with the UNVAC group at D43 pv (D15 pc) (Figure 11). At that time point, the CMI response in the SIVAC group was also significantly higher than in the VAC group (281 ± 315 and 131 ± 151 and IFNg-SCs per million of PBMC for the SIVAC and VAC groups, respectively, *p* = 0.041). At D56 pv, only the SIVAC group demonstrated a significantly higher level of IFNg-SCs compared with the UNVAC group. No cellular immune response toward the Finistere strain was detected in BALF during the post-challenge period, whatever the group.

## 4. Discussion

The objective of this study was to assess the hypothesis that a swIAV infection could affect the vaccine viremia, the post-vaccination immune response and the vaccine efficacy of an MLV1 in piglets, possibly through the production of large quantities of the anti-viral cytokine IFNa. Our results showed that, in the conditions of this study, a concomitant infection with the swIAV may actually interfere with an MLV1 vaccination at the systemic level by both delaying the vaccine viremia by one week and reducing the MLV1 genomic loads at the viremia peak by 10-fold. In SIVAC piglets, the PRRSV seroconversion was also delayed for a few days compared with VAC piglets, probably as the consequence of the later production of MLV1 antigens. Surprisingly, the swIAV infection conversely affected the cell-mediated immune response (CMI) against the PRRSV, tending to enhance the number of MLV1-specific IFNg-SCs during the post-vaccination period. This stronger CMI response in the SIVAC group was confirmed after the challenge with the PRRSV1 field strain 28 days after vaccination. Finally, however, the vaccine’s efficacy, evaluated by analyzing clinical and virological parameters following the infectious challenge, was not impacted by swIAV infection in our experimental conditions.

In SIVAC piglets, hyperthermia appeared at D1 pv and growth retardation started the first week of the experiment (D2 to D9 pv) with no respiratory symptoms; furthermore, swIAV nasal excretion was detected from D1 to D7 pv (or post-swIAV inoculation). These results were similar to those we previously described when inoculating the same H1N2 swIAV strain in unvaccinated SPF piglets [25], indicating that in the present study swIAV infection was not impacted by a concomitant MLV1 vaccination. As expected, the swIAV infection induced a transient IFNa production for four days, with high levels quantified as early as D1 pv. At D7 pv, the detection of the MLV1 viremia coincided with both IFNa clearance and the end of swIAV nasal excretion. Although the MLV1 replication was only temporarily inhibited during one week, these results supported those obtained by Brockmeier et al. who showed that the inoculation of piglets with an adenoviral vector that produced IFNa (Ad5-pIFNa) completely abolished the replication of an MLV2 as well as the post-vaccination immune response [16]. The two studies displayed a similar period of IFNa production but the different unit used for IFNa quantification precluded a comparison of IFNa concentrations. The incomplete inhibition of the MLV1 replication observed in our study may rely on different factors. Firstly, IFNa production following swIAV infection was possibly weaker than after Ad5-pIFNa inoculation. Secondly, as Brockmeier et al. applied simultaneous inoculations of Ad5-pIFNa and MLV2, the 6 h delay we applied between swIAV inoculation and the MLV1 vaccination might have played a role. However, as delayed and reduced viremia were similarly observed when the Ad5-pIFNa was administrated simultaneously or one day before the inoculation of a PRRSV2 field strain, this suggested that the delay between IFNa induction and the MLV replication might not be so decisive [31,32]. Thirdly, although similar vaccine doses were used, a different sensitivity to IFNa between MLV1 and MLV2 cannot be excluded. Lastly, the differences in the vaccine strain replication kinetics between MLVs [33] may also to be considered. Indeed, the Ingelvac PRRS MLV2 used by Brockmeier et al. showed a faster onset of viremia compared with the Porcilis PRRS MLV1 we used with 80% and 0% of viremic piglets at D3 pv for the MLV2 and the MLV1, respectively [33]. In our study, the slow replication of the MLV1 we used and consequently its slower host systemic spread might have protected from an IFNa effect a few viral particles left in tissues close to the vaccination site. Alternatively, as MLV strains are able to persist in tonsils or lymph nodes of non-viremic animals [34,35], it is likely that a low replication of the MLV1 in these tissues could have occurred during the phase of no viremia observed in the SIVAC group after vaccination. Later, these virions may have been able to relaunch the vaccine viremia once the IFNa had disappeared.

The swIAV infection could also have protected the MLV1 from a complete elimination from the host during the phase of no vaccine viremia observed until D7 pv in the SIVAC group. The role of IL10 in the swIAV infection is unclear but the early production of this cytokine was reported in the lungs of swIAV singly-infected pigs or swIAV and PRRSV co-infected pigs [36,37]. In this study, we also quantified a high IL10 gene expression in BALCs from the SIVAC group with a peak as early as D1 pv. IL10 is known to be an immunosuppressive cytokine with capability of downregulating the host immune responses and especially the IFNg T-helper 1 responses. IL10 was also shown to inhibit the expression of IFNa-induced genes [38]. Thus, the induction of IL10 production at an early stage after MLV1 vaccination may have contributed to the vaccine strain survival within the host during the phase of no vaccine viremia.

Unlike field PRRSV1 strains (including the Finistere strain) that were detected in BALF as early as D2 post-inoculation [27], the detection of MLV1 in lungs was late for both vaccinated groups. However, contrary to the blood, no delay in vaccine strain detection was observed in BALF between the SIVAC and VAC piglets. In addition, the MLV1 strain seemed to replicate at a higher level in the lungs of SIVAC piglets compared with VAC ones. For SIVAC pigs, the MLV1 genome detection started from D10 pv when IFNa levels were undetectable and the swIAV was almost no longer present in BALF. Here also, we can hypothesize that the high IL10 levels measured in BALCs could have provided a favorable environment for MLV1 replication in the lungs of SIVAC piglets.

It is interesting to mention that the observations we made with the H1N2 strain we used in this study may not be reproduced with other swIAV strains or subtypes depending on the cytokine response they induce. For example, we recently showed that infection with a H1N2 variant induced a stronger cytokine response including Tumor Necrosis Factor alpha (TNFa) production than infection with the parental H1N2 [25]. In vitro studies showed that TNFa, combined or not with IFN beta, markedly inhibited respiratory syncytial virus replication in human lung epithelial cells [39]. Considering the role of IFNa and maybe other cytokines in the interference of the swIAV with PRRS MLVs, we could hypothesize that swIAV strains inducing a higher cytokine production could result in an exacerbated interference with PRRSV vaccination. In addition, the endotracheal route used for swIAV inoculation as well as the dose administrated could have affected the results of this study. As reviewed by Van Reeth et Ma in 2013, an intranasal route or an endotracheal route using a lower swIAV dose lead to slower and lower viral load peaks in lungs, milder lung inflammation (lower levels of IFNa, IFNg and pro-inflammatory cytokines in BALF) and less influenza-specific symptoms [40]. The MLV1 vaccination route could additionally have impacted the results obtained in this study as it was demonstrated that intramuscular PRRSV vaccination induced a lower cell-mediated immune response than intradermal vaccination [5,41].

Apart from the role of IFNa or other cytokines, alternate mechanisms could contribute to the interference induced by one virus on the replication of another such as competition for cellular receptors or competition for molecular substrates required for replication. PRRSV1 and the swIAV may interact at several stages [42]; thus, additional mechanisms independent of the host anti-viral response may also be involved in the results obtained.

In our experimental set-up, our results showed an interference of swIAV infection on MLV vaccination in piglets. In children vaccinated with an anti-influenza MLV, the vaccine viral loads were lower in children with a co-detection of a non-influenza respiratory virus at the time of vaccination [43], suggesting that other MLVs could be also subjected to viral interference. More globally, the interference of influenza with other viral infections has been confirmed. Essaidi-Laziosi et al. recently demonstrated that the influenza virus interfered with respiratory syncytial virus replication in in vitro reconstituted airway epithelial tissues through a mechanism involving the anti-viral action of type I and type III IFNs [44].

In SIVAC piglets, in contrast to the delay we observed for blood PRRSV seroconversion, PRRSV antibody detection seemed to occur earlier in the lungs. These results could firstly be linked to the faster MLV1 replication in the lungs of SIVAC piglets. Another hypothesis could be that the host immune response to the swIAV infection in lungs associated with a recruitment of immune cells such as dendritic cells and γδ T cells [45] would have stimulated an earlier production of PRRSV antibodies. Interestingly, despite a high individual variability, the post-vaccination CMI against the PRRSV tended to be enhanced by the swIAV infection in the blood as in the lungs. Moreover, the gene expression of IFNg was upregulated in the BALCs of the SIVAC group. In this group, the balance between Th1-related IFNg and Th2-related IL10 could be in favor of the Th1 response as the gene expression upregulation was ten-fold higher for IFNg than for IL10. At the systemic level, we previously detected the H1N2 swIAV-specific CMI from D7 post-infection [25], which was earlier than the response generally induced against MLV1. In the SIVAC group, the recruitment of the immune cells involved in swIAV-specific CMI could thus have stimulated the anti-MLV1 CMI, explaining it was detected faster than in the VAC group especially in the lungs where both viruses were co-localized. The greater induction of CMI was then confirmed after the PRRSV1 challenge when a significantly higher IFNg-SC count was observed at D43 pv (D15 post-challenge) in SIVAC pigs compared with VAC pigs. Beyond its anti-viral effect, IFNa can also activate the adaptive cellular immune response by stimulating the dendritic cells that trigger antigen-specific T cell proliferation [46]. A correlation between the IFNa and IFNg responses was demonstrated after the co-administration of an IFNa-encoding plasmid and MLV2 [30]. Similarly, Brockmeier et al. showed an increase in IFNg-SCs when they administrated an Ad5-pIFNa simultaneously to a PRRSV2 field strain [31]. The enhanced PRRSV-specific CMI observed in SIVAC piglets after the PRRSV challenge was not sufficient to improve the control of the PRRSV viremia. Nevertheless, this better CMI could have at least counterbalanced the late PRRSV humoral response (linked to the delayed vaccine viremia) we measured in the SIVAC piglets.

In our experimental conditions, we demonstrated that swIAV infection altered the PRRSV1 MLV viremia and immune responses in piglets with no impact on vaccine efficacy. However, as discussed earlier, an interference on vaccine efficacy could be expected depending on the delay between swIAV infection and PRRSV MLV vaccination, the swIAV subtype, the PRRSV MLV strain, the routes and doses used for swIAV infection or the piglet immune status. We could expect that our study may pave the way for other studies to investigate the interference between PRRSV MLVs and swIAVs and also more globally between PRRSV MLVs and other swine viral infections that are frequently detected at the time of MLV vaccination. Better deciphering the interaction between PRRSV MLVs and swine viral infections seems to be essential for a better understanding of the poor efficacy of MLVs under field conditions and to improve their effectiveness.

## Data Availability

Not applicable.
